# QTL mapping, validation, and candidate gene predicting for maize plant architecture traits via bulked segregant analysis plus linkage analysis with F₂ population

**DOI:** 10.1186/s12870-026-08696-3

**Published:** 2026-04-14

**Authors:** Shiyu Wang, Yunhong Wang, Xihuan Li, Baoxin Ge, Chengfu Su

**Affiliations:** College of Agronomy, Qingdao Agricultural University, Qingdao, 266109 P. R. China

**Keywords:** Maize, Leaf angle, Tassel branch number, Plant height, BSA, QTL mapping, Candidate gene

## Abstract

Maize (Zea mays L.) is a globally important food crop whose plant architecture directly influences yield formation by regulating lodging resistance and photosynthetic efficiency, so deciphering the genetic mechanisms underlying plant architecture traits is essential for breeding high-yield varieties. In this study, maize inbred lines C144 and Su54—exhibiting significant differences in plant height (PH), tassel branch number (TBN), and leaf angle (LA)—were selected as parents; we first conducted preliminary QTL (Quantitative Trait Locus) mapping using BSA (bulked segregant analysis) to construct extreme phenotype bulks, then combined phenotypic data from the F₂ segregating population to build a genetic map with QTL IciMapping V4.2 software, and validated QTL loci via ICIM (inclusive composite interval mapping). The results revealed five validated major plant architecture-related QTLs: two for PH, *qPH5-1* on chromosome5 (phenotypic variation explained [PVE] = 13.75%, LOD (Logarithm of Odds) = 19.87) and *qPH9-1* on chromosome9 (PVE = 6.69%, LOD = 9.73); one for TBN, *qTBN5-1* on chromosome5 (PVE = 6.37%, LOD = 7.6); and two for LA, *qLA5-1* (penultimate leaf angle) on chromosome5 (PVE = 6.16%, LOD = 7.2) and *qLA5-2* (above-ear leaf angle) on chromosome5 (PVE = 9.74%, LOD = 11.69). Based on gene annotation and functional prediction within these QTL intervals, six candidate genes were identified: *Zm00001eb238970* and *Zm00001eb238980* (encoding P21-Arc proteins, subunits of the ARP2/3 complex) in *qPH5-*1; *Zm00001eb394120* (encoding a protein phosphatase 2 C) in *qPH9-1*; *Zm00001eb243000* (encoding an E2F transcription factor) in *qTBN5-1*; *Zm00001eb243750* (encoding a GTP/ATP-binding protein) in *qLA5-1*; and *Zm00001eb233650* (encoding a SUN family protein with Sad1/UNC domains) in *qLA5-2*. These novel QTL loci and candidate genes provide critical insights into the genetic regulatory network of maize plant architecture and can directly support molecular marker-assisted breeding to accelerate the development of high-yield and dense-tolerant maize varieties.

**Supplementary Information:**

The online version contains supplementary material available at 10.1186/s12870-026-08696-3.

## Introduction

Maize (Zea mays L.) is one of the most widely cultivated food and feed crops globally, and increasing its yield is of strategic significance for ensuring food security [1]. As a core agronomic trait determining maize yield, plant architecture directly regulates photosynthetic efficiency, lodging resistance, and adaptability to planting density. Therefore, “Ideal Plant Architecture (IPA) breeding” has become a key direction in modern maize breeding. Among the three core traits of maize plant architecture—plant height (PH), tassel branch number (TBN), and leaf angle (LA): PH affects lodging resistance and resource allocation [2, 3]; TBN determines pollen supply efficiency and the balance of photosynthate competition [4]; LA influences canopy light distribution and canopy photosynthetic efficiency [5–8]. Deciphering the genetic mechanisms of these traits and identifying key regulatory genes are of great value for promoting the application of molecular breeding technologies and accelerating the cultivation of high-yield, high-quality varieties.

PH, TBN, and LA are typical quantitative traits regulated by polygenic networks. QTL mapping, as a core tool for analyzing the genetic basis of quantitative traits, has been widely used in maize plant architecture research. For PH: Beavis et al. [9] first identified 16 PH QTLs using four F₂:₃ populations; Lima et al. [10] detected six related loci in an F₂ population; Wang et al. [11] identified 51 QTLs in recombinant inbred lines (RILs) using a high-density Bin marker map (16,769 markers); H Yang et al. [12] further screened six candidate genes in RIL and IBL populations and verified their homologous functions. For TBN: Berke and Rocheford [13] mapped three QTLs in a high-oil × low-oil maize F₂ population; Mickelson et al. [14] detected six loci in B73×Mo17 RILs using RFLP (Restriction Fragment Length Polymorphism)/SSR (Simple Sequence Repeat) markers; Tian et al. [4] fine-mapped *qTBN7* to a 110 Mb interval containing 14 candidate genes (including the known regulatory gene ramosa1). For LA: Jianbo Fei et al. [15] identified three regulatory genes on chromosomes 3 and 5; Mickelson et al. [14] detected nine QTLs in B73×Mo17 populations, with major loci on chromosomes 1 and 7 explaining 20.6% and 27.7% of phenotypic variation, respectively; Guo et al. [16] identified four QTLs using chromosome segment substitution lines, with a cumulative contribution rate of 53.3%.

Although significant progress has been made in QTL mapping of plant architecture traits, functional gene cloning remains limited. Among the cloned genes: *RPH1* affects PH by regulating microtubule extension direction [17]; *GIF1* participates in the regulation of meristem development [18]; TBN-related genes of the ramosa family [19–21] and *Tu1* [22] influence branch number by regulating the formation of branch meristems; LA regulatory genes *ZmTACI* [23], *UPA2* [24], and *bzrI* [25] alter leaf angle through cell elongation or transcriptional regulatory networks. These results indicate that there are still many unknown pathways in the genetic regulatory network of plant architecture development, and more key genes need to be identified through new strategies.

Bulked Segregant Analysis (BSA) rapidly locates target trait intervals by sequencing bulks of extreme phenotypes [26], and the QTL-seq method derived from high-throughput sequencing has become an efficient tool for gene mapping [27]. Inclusive Composite Interval Mapping (ICIM) significantly improves the accuracy and power of QTL detection by automatically selecting covariates and controlling background interference, making it particularly suitable for analyzing complex quantitative traits [28, 29]. However, most current studies rely on single populations or methods, leading to low QTL validation rates and insufficient mining of major loci.

In this study, maize inbred lines C144 and Su54—with significant differences in plant architecture traits—were used as parents. We adopted the strategy of “BSA-based preliminary mapping combined with F₂ population linkage analysis” and applied ICIM to map and validate QTLs for PH, TBN, and LA. The objectives were: (1) to identify stable major QTL loci; (2) to predict candidate genes and analyze their potential functions; (3) to provide theoretical basis and genetic resources for molecular breeding of maize plant architecture.

## Materials and methods

### Preliminary BSA mapping

#### Construction of preliminary mapping population and phenotypic investigation

Maize inbred lines C144 (low PH, more TBN, small LA) and Su54 (high PH, fewer TBN, large LA) with significant plant architecture differences were selected as parents. Phenotypic data (mean of three replicates under the same environment) for the parents included C144 with 159 cm PH, 8 TBN, and 12° LA, and Su54 with 250 cm PH, 3 TBN, and 54° LA. F₁ hybrids were obtained via artificial hybridization, and F₂ segregating populations were generated by self-pollination of F₁ for preliminary QTL mapping of plant architecture traits. At the grain filling stage (V12-V14 growth stage), phenotypic measurements of F₂ individuals were performed, including PH measured as the vertical distance from ground surface to tassel apex with 1 cm precision, TBN counted as the number of effective branches on the main spike axis, and LA determined as the angle between the penultimate leaf and stem with 1° precision; each trait was measured three times, and the mean value was used for subsequent analysis.

#### DNA extraction and construction of BSA extreme pools

For the construction of extreme DNA bulks, the F₂ population was ranked based on relative phenotypic values of PH, TBN, and LA, respectively. Plants with phenotypic values ranked in the top 5% (high-value bulk) and bottom 5% (low-value bulk) were selected to construct two contrasting bulks. Each bulk contained about 30 individuals to ensure sufficient phenotypic difference and population representativeness. Genomic DNA was extracted from these extreme individuals and parents (C144 and Su54) using a high-throughput plant genomic DNA extraction kit (Tiangen Biotech, Beijing, China). DNA quality was verified through two consecutive criteria: first, integrity was assessed via 1% agarose gel electrophoresis run at 120 V for 30 min to ensure no tailing or degradation of DNA bands, and second, concentration and purity were determined using a Qubit 4.0 Fluorometer (Thermo Fisher Scientific, Waltham, MA, USA) to confirm a DNA concentration of at least 50 ng/µL and an OD₂₆₀/OD₂₈₀ ratio ranging from 1.8 to 2.0. Qualified DNA from extreme individuals was mixed in equal amounts (2 µg per plant) to construct six extreme trait pools (PH high/low, TBN high/low, LA high/low) along with two parental pools (C144 and Su54), and all samples were stored at -20℃ for later use.

#### Sequencing and SNP marker development

Sequencing and SNP marker development were performed by Shijiazhuang Boruidi Biotechnology Co., Ltd. (http://www.molbreeding.com) using GenoBaits technology from Genotyping By Target Sequencing (GBTS) [30]. The workflow involved DNA fragmentation using a Covaris M220 Ultrasonic Disruptor (Covaris, Woburn, MA, USA) to generate 200–300 bp fragments, followed by library construction through end repair, 3’ adenylation, adapter ligation, size selection with AMPure XP beads (Beckman Coulter, Brea, CA, USA), PCR amplification with Barcode sequences, and library purification. Quality control was conducted using a Qubit 2.0 Fluorometer for initial concentration, an Agilent 2100 Bioanalyzer (Agilent Technologies, Santa Clara, CA, USA) for fragment distribution, and qPCR to confirm an effective concentration of at least 2 nM. Sequencing was carried out on the Illumina HiSeq X Ten platform with a depth of no less than 10×. Raw reads were filtered using fastp software [31] (v0.20.0, parameters: -n10 -q20 -u40 -l50) to remove adapter-containing, low-quality (Q < 20), and sequences shorter than 50 bp, yielding clean reads. Clean reads were aligned to the maize B73 reference genome (B73-REFERENCE-NAM-5.0, MaizeGDB) using the BWA-MEM algorithm (v0.7.17) [32]. SNP variants were detected using the UnifiedGenotyper module of GATK software (v3.5-0-g36282e4) [33] with filtering criteria including coverage depth ≥ 5×, SNP quality ≥ 30, minor allele frequency (MAF) ≥ 0.05, and missing rate ≤ 20%. Functional annotation of SNPs was performed using ANNOVAR software [34].

#### Preliminary BSA mapping

##### SNP-index association analysis

**Fig. 1:**
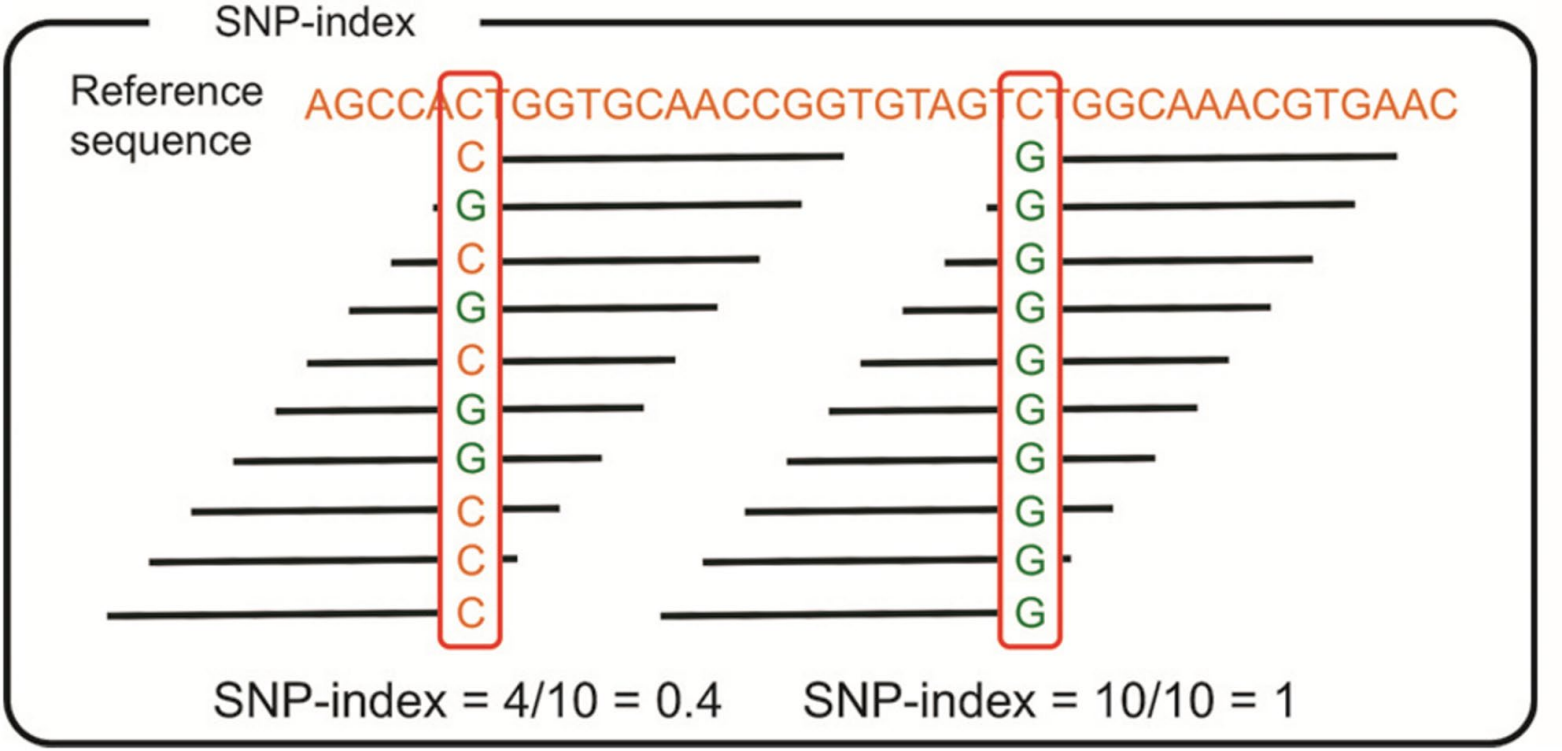
Calculation method of SNP-index analysis for preliminary mapping by BSA. Parental line 1 (carrying extreme trait A) was selected as the reference. The SNP-index (i.e., the frequency of single nucleotide polymorphisms) at marker loci filtered between the two parents was calculated for the two bulked progeny samples. Specifically, the SNP-index was assigned a value of 0 for marker loci completely identical to parental line 1, and 1 for those completely different from parental line 1

SNP-Index association analysis began with filtering SNPs to retain homozygous polymorphic SNPs between parents (C144 = aa, Su54 = AA) while excluding low-confidence sites with read support < 5 and multi-genotype sites. Next, SNP-Index values were calculated, where the SNP-Index of the high-value pool was defined as the frequency of the Su54 allele (A) and that of the low-value pool as the frequency of the C144 allele (a) (Fig. 1). Δ(SNP-Index) was then computed as the difference between the high-value pool and low-value pool SNP-Index values, and significance was determined via 1000 permutation tests to establish a 99% confidence threshold, with significant regions selected if Δ(SNP-Index) was close to 1, the high-value pool SNP-Index > 0.8, and the low-value pool SNP-Index < 0.2 [35].

##### Euclidean distance (ED) algorithm

**Fig. 2:**
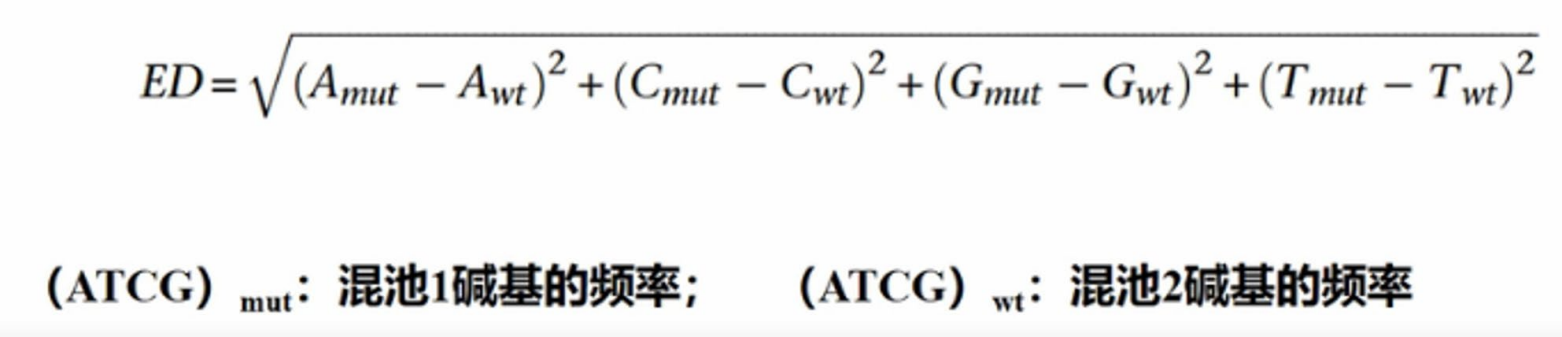
Formula of ED association method for preliminary mapping by BSA. (ATCG)_mut_ refers to the frequency of bases in the bulk 1; (ATCG)_wt_ refers to the frequency of bases in the bulk 2; A_mut_ refers to the frequency of adenine (A) in the mutant bulk; A_wt_ refers to the frequency of adenine (A) in the wild-type bulk; C_mut_ refers to the frequency of cytosine (C) in the mutant bulk; C_wt_ refers to the frequency of cytosine (C) in the wild-type bulk; G_mut_ refers to the frequency of guanine (G) in the mutant bulk; G_wt_ refers to the frequency of guanine (G) in the wild-type bulk; T_mut_ refers to the frequency of thymine (T) in the mutant bulk; T_wt_ refers to the frequency of thymine (T) in the wild-type bulk

The Euclidean Distance (ED) algorithm was implemented using the MMAPPR package, starting with calculating ED values based on the allele frequencies (f₁, f₂) of the two pools and sequencing depth (n) as shown in Fig. 2. To amplify significant signals, the original ED values were raised to the 4th power (ED⁴) as the association statistic. Local polynomial regression (LOESS) was then applied to fit the ED⁴ values and generate a genome-wide smooth curve, and the association threshold was set as the median plus 3 standard deviations (SD) of the fitted values. Finally, SNP sites were selected if the mutation frequency in the high-value pool was > 0.75 and the ED⁴ value exceeded the threshold [36].

### Construction and validation of segregating population

#### Population construction and phenotypic investigation

F₁ was obtained by crossing C144 as the female parent and Su54 as the male parent, and F₂ seeds were generated by self-pollination of F₁. In summer 2021, 544 F₂ individuals were planted at the Qingdao Agricultural University Laiyang Experimental Station (120°42′E, 36°54′N) with field management following local high-yield maize cultivation standards, including a row spacing of 60 cm, plant spacing of 25 cm, and regular irrigation and pest control. Phenotypic measurements were optimized based on the method described by Shi et al. [37], with PH measured at the grain filling stage as the vertical height from ground to tassel apex with 1 cm precision, TBN counted at the pollen shedding stage as the number of effective branches on the main spike axis, penultimate leaf angle (PLA) measured at the pollen shedding stage as the angle between the midrib of the penultimate leaf and the stem with 1° precision, and above-ear leaf angle (AELA) measured at the pollen shedding stage as the angle between the midrib of the first leaf above the ear and the stem with 1° precision. Phenotypic data were organized using Microsoft Excel 2019, and descriptive statistics (mean, standard deviation, coefficient of variation) and normality tests (Shapiro-Wilk test) were performed using IBM SPSS Statistics 27.

#### DNA extraction and regional genetic map construction

##### DNA extraction

Fresh leaves (~ 2 g) of F₂ individuals were collected at the jointing stage (V6-V8), frozen in liquid nitrogen immediately, and stored at -80℃. Genomic DNA was extracted using the CTAB method [38], which involved incubating the samples with 2% CTAB extraction buffer (containing 100 mmol/L Tris-HCl pH8.0, 20 mmol/L EDTA, 1.4 mol/L NaCl, and 2% CTAB) at 65℃ for 1 h, followed by protein removal via chloroform-isoamyl alcohol (24:1) extraction, DNA precipitation with isopropanol, two washes with 70% ethanol, air-drying, and dissolution in TE buffer (10 mmol/L Tris-HCl pH8.0, 1 mmol/L EDTA). Qualified DNA samples were stored at -20℃.

##### Primer screening and polymorphism verification

Based on the BSA preliminary mapping results, SSR primers were screened from the MaizeGDB database (https://www.maizegdb.org) and an SSR primer library independently developed by Sichuan Agricultural University [39] within the candidate intervals of chromosome 5 (85–101 Mb, 160–209 Mb) and chromosome 9 (19–60 Mb, 88–119 Mb). Additionally, 10 SSR markers were selected outside these intervals as references to cover the full length of chromosomes 5 and 9. All primers were synthesized by Sangon Biotech (Shanghai) Co., Ltd. with PAGE purification. Using parental DNA (C144 and Su54) as templates for PCR amplification, polymorphic primers were screened via 8% polyacrylamide gel electrophoresis. Polymorphic SSR primers were selected according to multiple strict criteria: (1) primers showed high polymorphism information content (PIC > 0.5); (2) primer length ranged from 18 to 25 bp, GC content ranged from 40% to 60%, and annealing temperature was optimized between 53 ℃ and 60 ℃ to ensure stable amplification; (3) PCR products showed clear target bands with high amplification efficiency and good repeatability; (4) stable and clear polymorphism between the two parental lines. A total of 38 primers with clear polymorphism were obtained for F₂ population genotyping. The detailed information of polymorphic SSR markers, including marker name, chromosome location, forward and reverse primer sequences, and annealing temperature, is listed in Supplementary Table S1.

##### PCR amplification and genotyping

The PCR reaction system had a total volume of 10 µL, consisting of 5 µL 2×Taq Plus MasterMix (Dye) (Kangwei Century, Jiangsu, China), 1 µL each of forward and reverse primers (10 µmol/L), 1 µL genomic DNA (50 ng/µL), and 2 µL ddH₂O. The amplification program included pre-denaturation at 94℃ for 2 min, followed by 35 cycles of denaturation at 94℃ for 30 s, annealing at 54–64℃ (adjusted according to primer Tm value) for 30 s, and extension at 72℃ for 30 s, with a final extension at 72℃ for 2 min. Amplification products were separated by 8% non-denaturing polyacrylamide gel electrophoresis in 1×TBE buffer at 120 V for 180 min, then visualized via silver staining: samples were fixed in a solution of anhydrous ethanol: distilled water: glacial acetic acid (20:20:1) for 15 min, stained in 0.2% AgNO₃ solution in the dark for 10 min, developed in a solution of 1.5% NaOH plus 1% formaldehyde until bands were clear, and rinsed with tap water to terminate the reaction before photographing with a gel imaging system. Genotypes were recorded based on parental band differences: consistent with Su54 as “0”, consistent with C144 as “2”, heterozygous as “1”, and missing data as “.“, with results entered into Excel for linkage analysis.

##### Regional genetic map construction

Regional genetic maps of chromosomes 5 and 9 were constructed using the “Map” module of QTL IciMapping V4.2 with parameters set as follows: population type as F₂, genetic distance converted using the Kosambi function, step size of 1 cM, and LOD threshold determined via 1000 permutation tests (α = 0.05).

#### QTL validation

Combining the phenotypic data of four plant architecture traits from the F₂ population with the regional genetic map, QTL mapping was performed using the “ICIM-ADD” module of QTL IciMapping V4.2. Parameters were set as a scan step of 1 cM, significance level α = 0.05, and LOD threshold determined via 1000 permutation tests for each trait. QTLs were named following the rule: starting with “q”, followed by the trait abbreviation (PH, TBN, LA), chromosome number, and serial number, such as *qPH5-1* representing the first PH QTL on chromosome 5.

### Candidate gene prediction

Based on the validated QTL intervals, the physical maps of chromosomes 5 and 9 (B73-REFERENCE-NAM-5.0 version) were obtained from the MaizeGDB database. The physical position of the QTL peak was estimated using the linear interpolation method [40], and the candidate interval was defined as 200 kb on each side of the peak, totaling 400 kb. All gene annotation information (including gene ID, functional description, and protein domain) within the candidate interval was extracted using the JBrowse tool of MaizeGDB, and the expression levels of target genes in plant architecture-related tissues (internodes, shoot apical meristem, young leaf, tassel) were analyzed using the qTeller platform (https://qteller.maizegdb.org/) [41]. Candidate genes were selected if they had high FPKM values and functional annotations related to plant architecture regulation, and their functions were finally verified via alignment with the NCBI (National Center for Biotechnology Information) database.

## Results and analysis

### Preliminary BSA mapping results

#### Construction of extreme bulks

Extreme individuals were selected from the F₂ population based on the top 5% and bottom 5% of phenotypic values for plant architecture traits to construct BSA bulks: 27 individuals each for extreme low (≤ 3) and high (≥ 12) TBN; 24 and 46 individuals for extreme small (≤ 15°) and large (≥ 45°)LA, respectively; 23 and 33 individuals for extreme high (≥ 280 cm) and low (≤ 160 cm) PH, respectively. Additionally, 5 plants each from parents C144 and Su54 were selected to construct parental bulks as controls. Individual DNA samples were qualified after 1% agarose gel electrophoresis (for integrity) and Qubit quantification (concentration ≥ 50 ng/µL, OD₂₆₀/OD₂₈₀ ratio = 1.8-2.0). Equal amounts of qualified DNA were mixed to form 8 bulks (6 extreme trait bulks + 2 parental bulks). Details of bulk construction are provided in Table 1.

**Table 1:** Statistics of bulked samples for plant height (PH), tassel branch number (TBN), and leaf angle (LA) traits in F₂ population derived from cross between maize inbred lines C144 and Su54

	Extreme low TBN	Extreme high TBN	Extreme small LA	Extreme large LA	Extreme high PH	Extreme low PH	Su54	C144
Quantity	27	27	24	46	23	33	5	5
Sample number	1	2	3	4	5	6	7	8

#### Sequencing data quality and SNP marker development

A total of 316,916,508 raw reads were generated by sequencing. After filtering with fastp (parameters: -n 10 -q 20 -u 40 -l 50), 316,885,516 clean reads were obtained with a Q30 ratio ≥ 92.3% and an average sequencing depth of 12.5×. The alignment rate to the maize B73 reference genome (B73-REFERENCE-NAM-5.0) was 98.7%, indicating that the sequencing data quality met the requirements for subsequent analysis (Table 2). Through GATK variant calling and filtering (coverage depth ≥ 5×, SNP quality ≥ 30, MAF ≥ 0.05, missing rate ≤ 20%), 264,630 initial SNP sites were identified. The SNP missing rate for all 8 bulks was < 3% (Table 3), and the final number of valid SNPs per bulk ranged from 263,089 to 264,207, which were used for subsequent association analysis. The raw sequencing data generated in this study have been deposited in the NCBI SRA (Sequence Read Archive) under the accession number PRJNA1436033.

**Table 2:** Quality statistics of sequencing data for bulked samples of PH, TBN, and LA traits in F₂ population from C144 × Su54 maize inbred lines cross

Sample	Raw Reads Number	Clean Reads Number	Raw Bases(bp)	Clean Bases(bp)	Effective Rate(%)	Q20(%)	Q30(%)
1	53,725,964	53,716,780	8,058,894,600	7,935,015,546	98.46	97.80	88.38
2	53,837,602	53,832,878	8,075,640,300	7,832,110,638	96.98	97.91	89.12
3	56,359,550	56,350,390	8,453,932,500	8,239,180,562	97.46	97.90	89.22
4	66,212,288	66,204,932	9,931,843,200	9,691,301,112	97.58	97.80	89.40
5	62,008,256	62,002,368	9,301,238,400	9,114,400,244	97.99	97.79	88.85
6	55,571,620	55,566,634	8,335,743,000	8,136,762,568	97.61	97.91	89.05
7	105,213,856	66,204,932	15,782,078,400	15,344,135,502	97.23	97.95	89.66
8	94,122,776	94,113,140	14,118,416,400	13,826,278,344	97.93	97.89	88.90

The columns in this table are defined as follows: Sample refers to the ID/name of the bulked sample pool; Raw Read Number denotes the count of raw sequencing reads; Clean Read Number represents the count of clean sequencing reads after quality filtering; Raw Base (bp) indicates the raw sequencing data output, calculated as the product of the number of raw reads and their length, with units in base pairs (bp); Clean Base (bp) stands for the effective data volume obtained after filtering, which equals the total length of clean reads, in base pairs (bp); Effective Rate (%) is the ratio of clean data to raw data after filtering, expressed as a percentage; Q20 and Q30 refer to the percentage of bases with Phred quality scores greater than 20 and 30, respectively, among all bases in the clean data

**Table 3:** Statistics of SNP loci in bulked samples for PH, TBN, and LA traits in F₂ population from C144 × Su54 maize cross

Sample	NA_ number	NA_ rate(%)	Het_alt_ number	Het_alt_ rate(%)	Hom_alt_ number	Hom_alt_ rate(%)	Ref number	Ref rate(%)
1	615	0.23	78,796	29.85	53,476	20.25	131,743	49.9
2	513	0.19	80,055	30.31	52,624	19.92	131,438	49.77
3	653	0.25	79,535	30.13	52,933	20.05	131,509	49.82
4	577	0.22	79,963	30.28	53,004	20.07	131,086	49.64
5	423	0.16	81,922	31.01	51,926	19.65	130,359	49.34
6	495	0.19	81,517	30.86	52,253	19.78	130,365	49.36
7	1451	0.55	3913	1.49	98,070	37.26	161,196	61.25
8	1541	0.58	3820	1.45	92,072	35	167,197	63.55

The columns in this table are defined as follows: Sample refers to the ID/name of the bulked sample pool; NA_number denotes the number of missing loci; NA_rate represents the missing rate, calculated as the ratio of the number of missing loci to the total number of loci; Het_alt_number indicates the number of heterozygous alternative loci; Het_alt_rate stands for the heterozygous alternative rate, which equals the ratio of the number of heterozygous alternative loci to the total number of non-missing loci; Hom_alt_number refers to the number of homozygous alternative loci; Hom_alt_rate represents the homozygous alternative rate, calculated as the ratio of the number of homozygous alternative loci to the total number of non-missing loci; Ref_number denotes the number of reference-consistent loci; Ref_rate stands for the reference-consistent rate, which is the ratio of the number of reference-consistent loci to the total number of non-missing loci

#### Preliminary BSA mapping of target traits

**Fig. 3:**
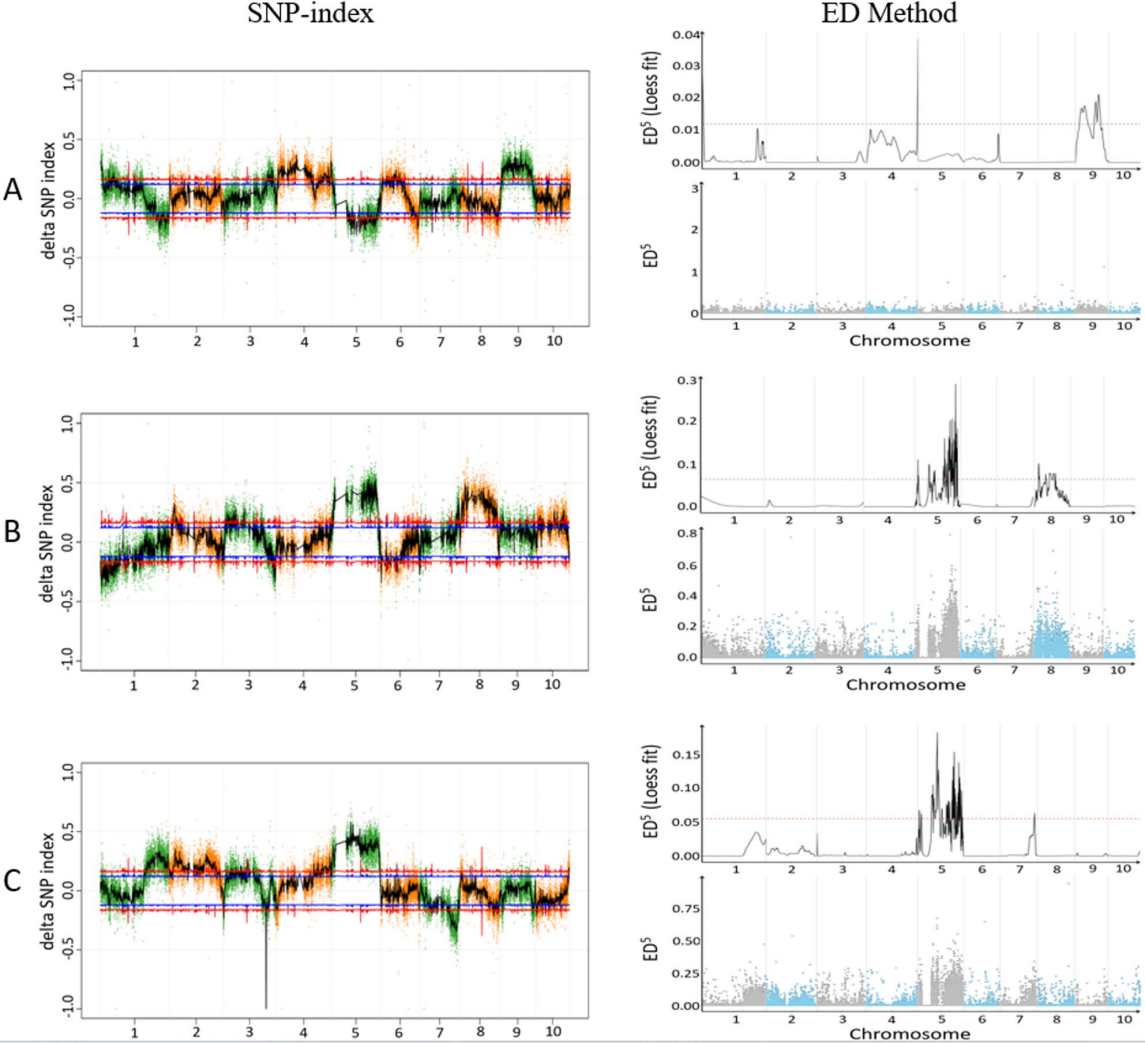
Results of SNP-index analysis and ED method for bulked samples of PH, TBN, PLA traits in F₂ population. **A**: Maize plant height trait; **B**: Maize tassel branch number trait; **C**: Maize penultimate leaf angle trait. In the Manhattan plot of SNP-index: The x-axis represents chromosomes, and the y-axis represents Δ(SNP-index) values. Each point indicates the position and corresponding Δ(SNP-index) value of an individual SNP. The lines denote the average Δ(SNP-index) value of all SNPs within each sliding window (1000 kb window size with a 500 kb step), reflecting the genome-wide trend of Δ(SNP-index) values. Blue line: 95% confidence level; Red line: 99% confidence level. In the ED association dot plot: The x-axis shows chromosome names. Dots represent the ED value of each SNP locus, and the line plot shows fitted ED values. A higher ED value indicates a stronger association at the locus. The blue shadow area indicates the mapped interval

Joint mapping was performed using Index analysis and ED algorithm, with intersection regions of the two methods considered as candidate intervals (Fig. 3). For PH: Index analysis detected 146 associated regions (total length 449.5 Mb), while ED analysis identified 4 regions (total length 81.75 Mb). The intersection revealed two candidate intervals on chromosome 9 (19.1–59.8 Mb and 88.4-118.6 Mb) containing 45 significant SNPs, suggesting major QTLs might be located on this chromosome. For TBN: Index analysis detected 401 associated regions (total length 345.75 Mb), and ED analysis identified 28 regions (total length 82.65 Mb). The intersection indicated a candidate interval on chromosome 5 (160.5–201 Mb) with 295 significant SNPs, implying it as the major QTL interval for TBN. For LA: Index analysis detected 139 regions (total length 531 Mb), and ED analysis identified 33 regions (total length 75.82 Mb). The intersection determined two candidate intervals on chromosome 5 (84.9-100.5 Mb and 174-210.5 Mb) containing 466 SNPs, which were inferred as major QTL intervals for LA.

### Validation of segregating population and QTL mapping results

#### Phenotypic analysis of plant architecture traits in F₂ population

**Fig. 4:**
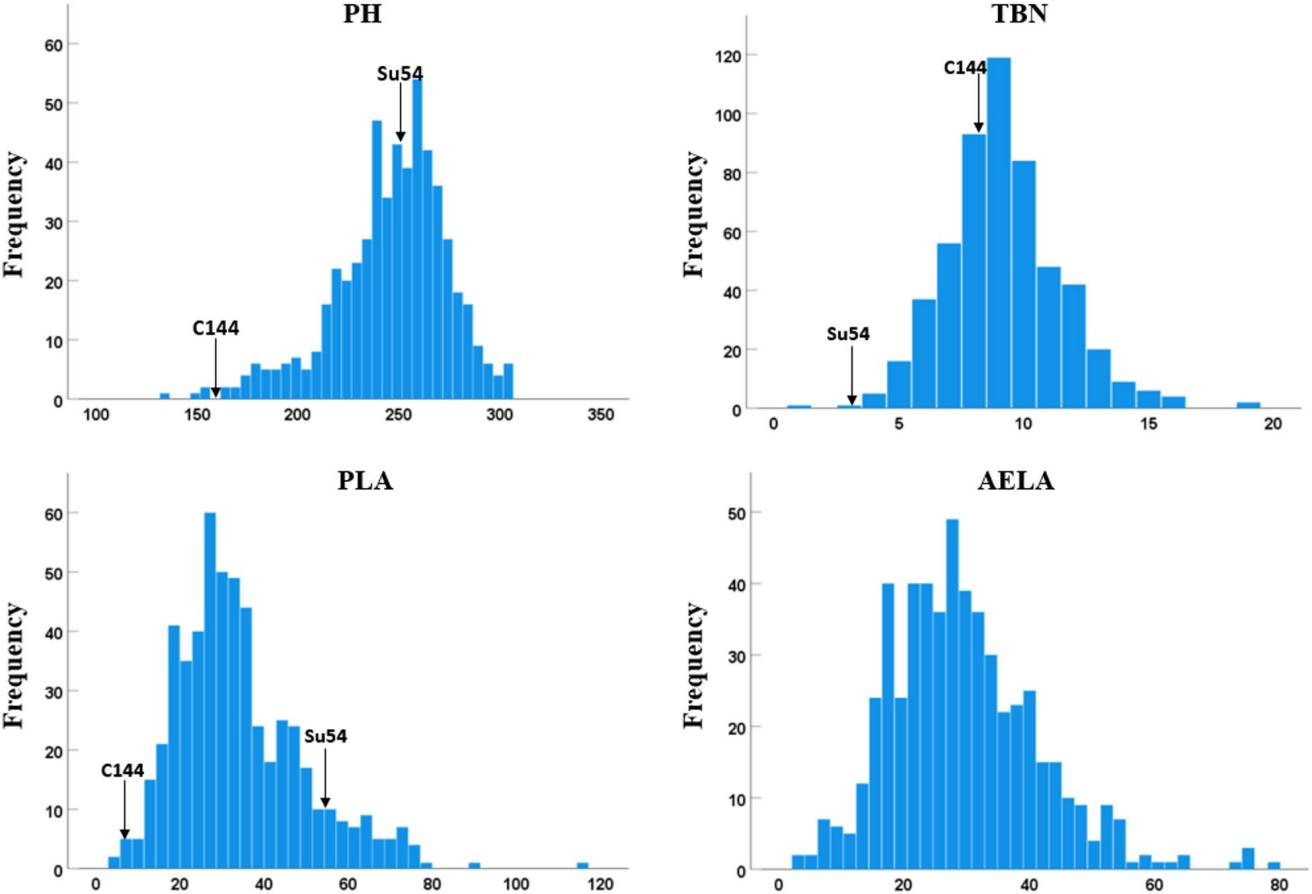
Frequency distribution of PH, TBN, PLA, and AELA traits in F₂ population derived from cross between maize inbred lines C144 and Su54. This figure displays the frequency distribution plots of PH, TBN, PLA, and AELA traits in the F₂ population derived from the cross between maize inbred lines C144 and Su54. Data for PH, TBN, and PLA of parental lines C144 and Su54 are marked with arrows in the plots

Phenotypic statistics for four plant architecture traits in 544 F₂ individuals are shown in Table 4: PH ranged from 134.00 to 305.00 cm (mean = 246.28 cm, CV = 11.38%); TBN ranged from 1 to 19 (mean = 9.18, CV = 25.31%); the PLA ranged from 5° to 115° (mean = 34.16°, CV = 44.44%); the AELA ranged from 3° to 78° (mean = 29.36°, CV = 40.42%). Shapiro-Wilk test indicated all traits followed a normal distribution (*P* > 0.05), and significant transgressive segregation was observed, suggesting these traits are controlled by multiple genes and suitable for QTL mapping (Fig. 4).

**Table 4:** Descriptive statistics of PH, TBN, penultimate leaf angle (PLA), and above-ear leaf angle (AELA) traits in F₂ population derived from C144 × Su54 maize inbred lines

Traits	Min	Max	Mean	SD	Var	CV(%)
PH(cm)	134.00	305.00	246.28	28.03	785.69	11.38
TBN	1	19	9.18	2.32	5.40	25.31
PLA(°)	5	115	34.16	15.18	230.51	44.44
AELA(°)	3	78	29.36	11.87	140.83	40.42

#### Construction of regional genetic map

**Fig. 5:**
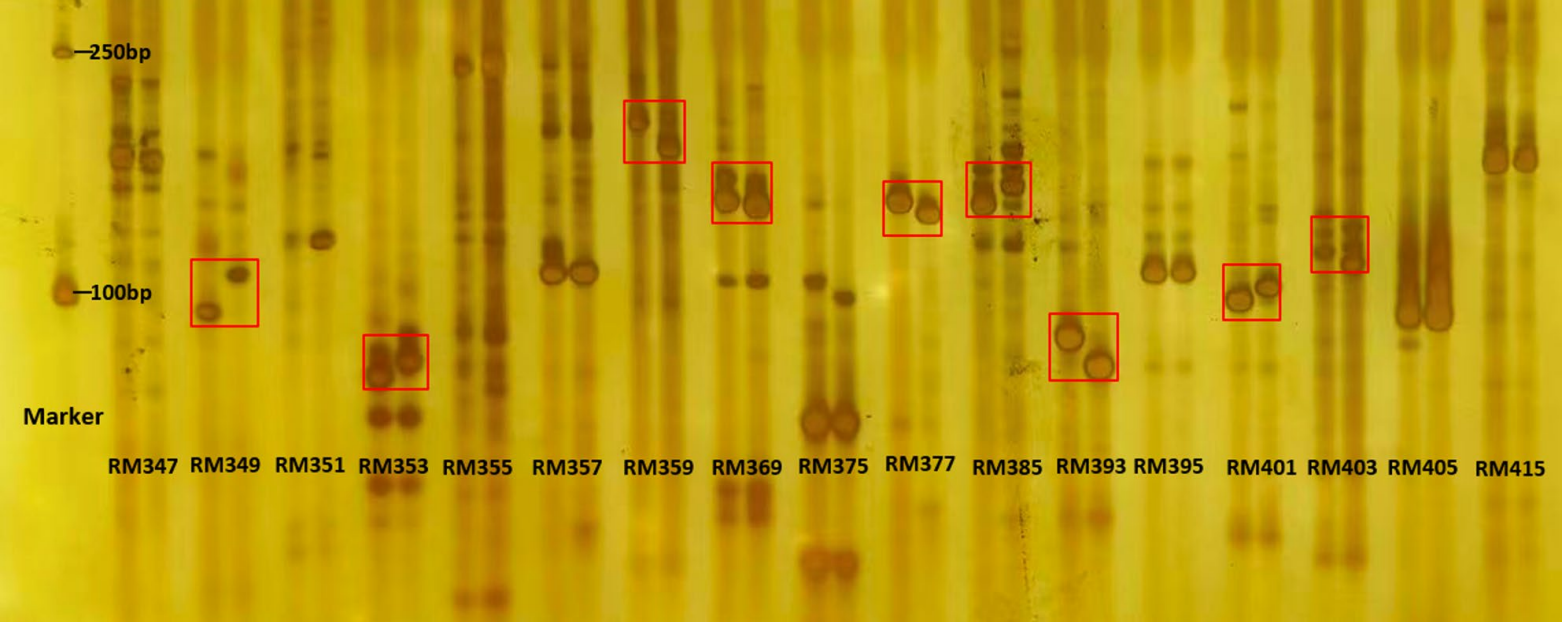
Screening of polymorphic SSR primers between parental lines C144 and Su54. Polymorphic primers (marked with red boxes) are selected for subsequent genotyping of F₂ population

**Fig. 6:**
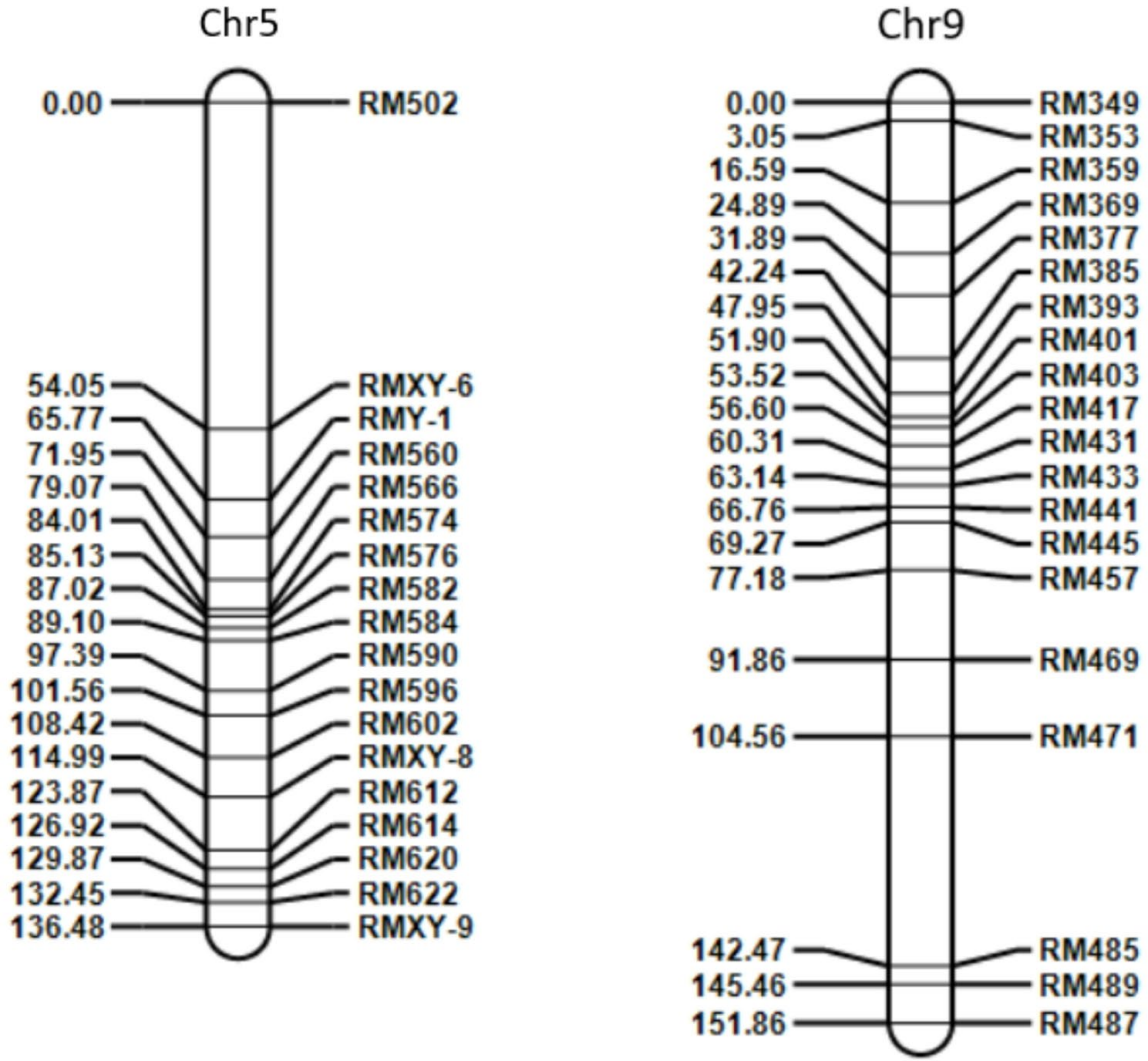
Segment genetic map of chromosomes 5 and 9 constructed by QTL IciMapping V4.2 software using F₂ population derived from cross between C144 and Su54

Based on the candidate intervals of chromosomes 5 (85-100.5 Mb, 160.5–201 Mb) and 9 (19.1–59.8 Mb, 88.4-118.6 Mb) from preliminary BSA mapping, SSR primers were screened and verified for polymorphism using parental DNA (typical results shown in Fig. 5). A total of 38 stable polymorphic primers were obtained (18 for chromosome 5, 20 for chromosome 9). Genetic maps were constructed using the “Map” module of QTL IciMapping V4.2. The results (Fig. 6) showed that the genetic map of chromosome 5 was 136.48 cM long with an average marker distance of 7.58 cM; the map of chromosome 9 was 151.86 cM long with an average distance of 7.59 cM. Marker order exhibited good collinearity with the physical map (collinearity coefficient > 0.95).

#### QTL mapping results

**Fig. 7:**
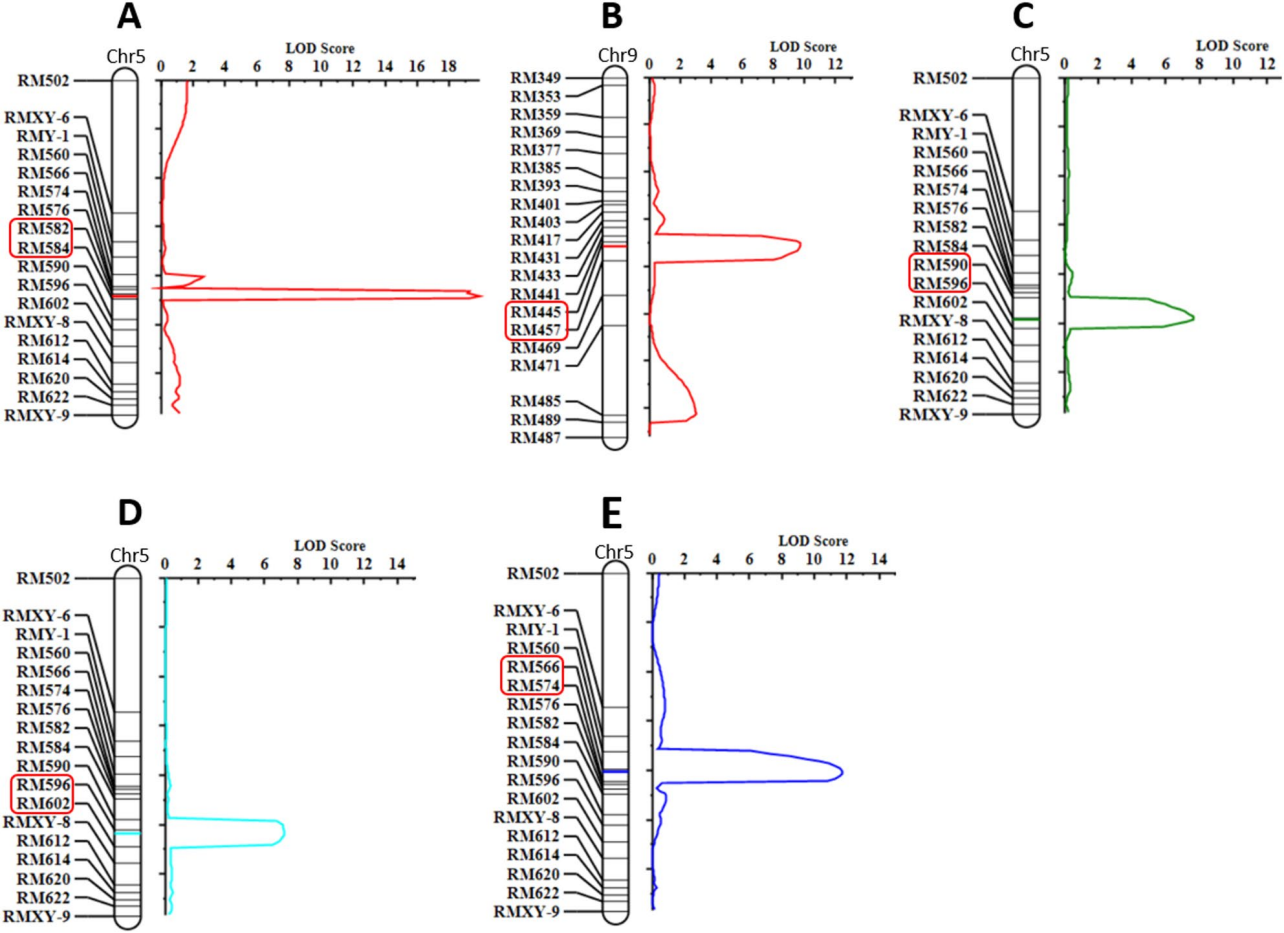
QTL mapping results for PH, TBN, PLA, and AELA traits in F₂ population (C144×Su54) using inclusive composite interval mapping (ICIM). Panels **A** and **B** represent QTL loci for PH on chromosomes 5 and 9 respectively, panel **C **shows TBN QTL on chromosome 5, panel **D **depicts PLA QTL on chromosome5, and panel **E **presents AELA QTL on chromosome5. In this multi-panel figure, the left side lists polymorphic primer names, the right side uses the x-axis to denote LOD scores, the colored horizontal lines on chromosomes indicate the positions of QTL loci for each trait, and the marker names on both sides of each identified QTL are marked with red rectangular boxes

Combining phenotypic data of four plant architecture traits from the F₂ population with the regional genetic map, QTL mapping was performed using the “ICIM-ADD” module of QTL (LOD threshold was 2.74 determined by 1000 permutation tests). Five major QTLs controlling maize plant architecture traits were detected (Table 5; Fig. 7), each showing independent and significant genetic effects.

**Table 5:** QTL mapping results for PH, TBN, PLA, and AELA traits using inclusive composite interval mapping (ICIM) in F₂ mapping population

Traits	QTL	Genetic Position/cM	Flanking Markers	Physical Range/Mb	LOD score	PVE/%	ADD	DOM	Ref.
PH	*qPH5*-1	88	RM582-RM584	146.7-153.7	19.87	13.75	-15.31	-0.10	Li et al. [42], (130-178 Mb) Du et al. [43], (147.4-175 Mb)
	*qPH9*-1	71	RM445-RM457	130.3–143	9.73	6.69	10.58	2.57	
TBN	*qTBN5*-1	98	RM590-RM596	172.8-175.5	7.60	6.37	0.87	0.38	Cong, et al. [44], (169.2-175.8 Mb)
PLA	*qLA5*-1	103	RM596-RM602	175.5-178.9	7.20	6.16	-5.62	1.61	
AELA	*qLA5*-2	80	RM566-RM574	89.4–95.4	11.69	9.74	-5.08	-2.03	

Ref. Results of previous studies

*ADD Estimated additive effect, DOM Estimated dominance effect*

For PH: Two major QTLs (*qPH5-1* and *qPH9-1*) were detected on chromosomes 5 and 9, collectively explaining 20.44% of phenotypic variation. *qPH5-1* was located in the interval 146.7-153.7 Mb on chromosome 5, explaining 13.75% of variation with an LOD score of 19.87 and an additive effect of -15.31. *qPH9-1* was located in the interval 130.3–143 Mb on chromosome 9, explaining 6.69% of variation with an LOD score of 9.73 and an additive effect of 10.58.

For TBN: One major QTL (*qTBN5-1*) was detected in the interval 172.8-175.5 Mb on chromosome 5, explaining 6.37% of variation with an LOD score of 7.6 and an additive effect of 0.87. This locus overlapped with the preliminary BSA interval (160.5–201 Mb), verifying its role as a key major genetic locus controlling TBN.

For LA: Two major QTLs were detected on chromosome 5, regulating the PLA and AELA, collectively explaining 15.90% of phenotypic variation. *qLA5-1* (PLA, interval 175.5-178.9 Mb) explained 6.16% of variation with an LOD score of 7.2 and an additive effect of -5.62. *qLA5-2* (AELA, interval 89.4–95.4 Mb) explained 9.74% of variation with an LOD score of 11.69 and an additive effect of -5.08. Both QTLs were within the preliminary BSA intervals (85-100.5 Mb and 174-208.4 Mb), confirming their major genetic effects.

### Candidate gene prediction and functional annotation

**Fig. 8:**
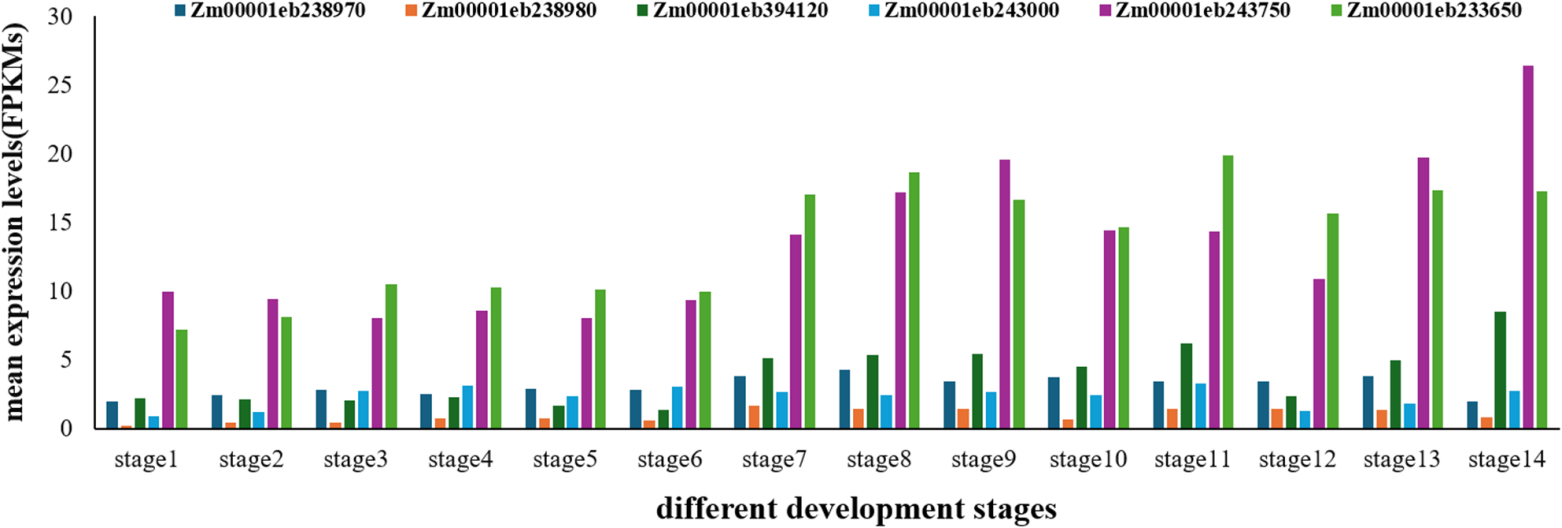
Histogram of average expression levels (FPKMs) of candidate genes in plant architecture-related tissues (different internodes, shoot apical meristem, young leaves, tassels) at different stages (as shown in Stelpflug et al. [41]. Different Colors Represent Different Candidate Genes, and Column Height Indicates Expression Level. stage1: Nonpollinated_internode_0_DAP; stage2: Nonpollinated_internode_6_DAP; stage3: Nonpollinated_internode_12_DAP; stage4: Nonpollinated_internode_18_DAP; stage5: Nonpollinated_internode_24_DAP; stage6: Nonpollinated_internode_30_DAP; stage7: V5_First_elonagated_internode; stage8: V9_Fourth_elongated_internode; stage9: V1_4D_PE_Stem_SAM; stage10: V3_Stem_and_SAM; stage11: V5_Shoot_tip; stage12: V5_Bottom_of_transition_leaf; stage13: V7_Bottom_of_transition_leaf; stage14: V13_Immature_tassel

Based on the physical positions of the five QTL intervals (B73-REFERENCE-NAM-5.0), a total of 44 protein-coding genes were extracted within 400 kb on either side of each QTL peak (Supplementary Table 2). Combined with functional annotations from the MaizeGDB database, tissue expression patterns from the qTeller platform, and literature reports, six core candidate genes were finally selected (Table 6). For PH-related candidate genes: Three genes were identified in *qPH5-1* and *qPH9-1* intervals. *Zm00001eb238970* and *Zm00001eb238980* (located in *qPH5-1*) encode the ARPC3 subunit of the ARP2/3 complex, which participates in cell elongation by regulating actin cytoskeleton dynamics [45–51] and is highly expressed in shoot apical meristems and internodes (Fig. 8). *Zm00001eb394120* (located in *qPH9-1*) encodes a protein phosphatase 2 C, a ortholog of the Arabidopsis POL gene, which regulates plant growth by maintaining the balance between proliferation and differentiation of meristematic stem cells [52]. For TBN-related candidate gene: *Zm00001eb243000 *(located in *qTBN5-1*) encodes an E2F transcription factor, a core regulator of cell cycle and division [53].`For LA-related candidate genes: Two genes were identified in *qLA5-1* and *qLA5-2* intervals. *Zm00001eb243750* (located in *qLA5-1*) encodes a GTP/ATP-binding protein; GO annotations suggest it may participate in chloroplast organization, cell division, and apical meristem development, implying it regulates LA by affecting pulvinus cell growth. *Zm00001eb233650* (located in *qLA5-2*) encodes a SUN family protein containing the Sad1/UNC domain, a core component of the LINC complex that participates in plant morphogenesis by regulating cell division and elongation [54–56], and is continuously highly expressed during leaf development (Fig. 8). Table 7 shows mean expression values (FPKMs) of each candidate gene in different stalk related tissues at different stages stage1 to stage14 denoted in the foot note of Table 7.

**Table 6:** Candidate genes predicted at five QTL loci associated with plant architecture traits based on QTL mapping results

Gene ID	QTL	Start(bp)	End(bp)	Length(bp)	Annotation
*Zm00001eb238970*	*qPH5*-1	149,845,020	149,846,651	1,631	P21-Arc
*Zm00001eb238980*	*qPH5*-1	149,890,028	149,890,930	902	P21-Arc
*Zm00001eb394120*	*qPH9*-1	132,988,328	132,996,488	8,160	Protein phosphatase 2 C
*Zm00001eb243000*	*qTBN5*-1	173,168,577	173,173,402	4,825	E2F transcription factor; core regulator of G1/S cell cycle transition
*Zm00001eb243750*	*qLA5*-1	176,358,316	176,362,215	3,899	A multifunctional protein with GTPase and hydrolase activity.
*Zm00001eb233650*	*qLA5*-2	90,721,578	90,727,950	6,372	Sad1 / UNC-like C-terminal

**Table 7:** Average expression levels (FPKMs) of candidate genes in plant architecture-related tissues (internodes, shoot apical meristem, young leaves, and tassels) at different developmental stages

	stage1	stage2	stage3	stage4	stage5	stage6	stage7	stage8	stage9	stage10	stage11	stage12	stage13	stage14
*Zm00001eb238970*	1.98	2.41	2.79	2.48	2.86	2.78	3.81	4.28	3.45	3.70	3.39	3.40	3.80	1.98
*Zm00001eb238980*	0.24	0.39	0.43	0.73	0.76	0.59	1.65	1.43	1.41	0.66	1.41	1.44	1.32	0.80
*Zm00001eb394120*	2.23	2.12	2.02	2.29	1.63	1.34	5.14	5.36	5.42	4.47	6.22	2.37	4.93	8.46
*Zm00001eb243000*	0.90	1.20	2.72	3.15	2.34	3.03	2.68	2.40	2.67	2.46	3.30	1.30	1.85	2.73
*Zm00001eb243750*	9.94	9.44	8.01	8.60	8.01	9.32	14.10	17.19	19.55	14.43	14.29	10.85	19.68	26.34
*Zm00001eb233650*	7.15	8.11	10.51	10.22	10.13	9.95	16.98	18.59	16.64	14.63	19.82	15.61	17.29	17.25

*FPKMs* fragments per kb exon model per million mapped fragments。

stage1: Nonpollinated_internode_0_DAP; stage2: Nonpollinated_internode_6_DAP

stage3: Nonpollinated_internode_12_DAP; stage4: Nonpollinated_internode_18_DAP

stage5: Nonpollinated_internode_24_DAP; stage6: Nonpollinated_internode_30_DAP

stage7: V5_First_elonagated_internode; stage8: V9_Fourth_elongated_internode

stage9: V1_4D_PE_Stem_SAM; stage10: V3_Stem_and_SAM

stage11: V5_Shoot_tip; stage12: V5_Bottom_of_transition_leaf

stage13: V7_Bottom_of_transition_leaf; stage14: V13_Immature_tassel

## Discussion

Maize, as the largest food crop in China, its yield improvement holds significant strategic importance for ensuring national food security. Since Donald proposed the concept of “ideal plant architecture” for crops in 1968 [57], dissecting the genetic mechanisms of plant architecture traits and mining key regulatory QTLs and genes have become core approaches for breeding high-yield maize varieties. Currently, in the field of plant genetics, multiple maize plant architecture-related QTLs have been mapped using different populations and marker technologies: Xing et al. [58] fine-mapped a major PH QTL to a 1632 bp interval on chromosome 1 using a recombinant inbred line (RIL) population and SSR markers; PAN et al. [59] detected 86 PH QTLs via joint linkage mapping (JLM) using a ROAM population (1887 plants) and 56,110 SNP markers, among which *qPH3* was narrowed down to a 600 kb interval containing three candidate genes; Upadyayula et al. [60] mapped a tassel branch number-related QTL near the *ramosa1* gene on chromosome 7; Ding et al. [61] detected 14 LA QTLs in two environments based on a four-way cross population and verified four of these loci using near-isogenic lines. These studies provide important references for dissecting the genetic network of maize plant architecture, but differences in genetic backgrounds, population sizes, and detection methods may lead to variations in QTL mapping results. Notably, this study conducted a detailed comparative analysis of QTL mapping results with previous relevant studies by reviewing multiple previous literatures. The *qPH5-1* locus (physical interval: 146.7-153.7 Mb) identified in this study overlaps with the locus reported by Du et al. (2021) (147.4-175Mb) [43] and the locus reported by Li et al. (2016) (approximately 130-178Mb) [42], while the *qTBN5-1* locus overlaps with the locus reported by Cong et al. (2017) (approximately 169.2-175.8Mb) [44]. These results suggest that *qPH5-1* and *qTBN5-1* can be repeatedly detected under different genetic backgrounds, showing stable heritability. Compared with previous studies, this study has confined *qPH5-1* and *qTBN5-1* to smaller physical intervals, with higher mapping accuracy. In addition, three new QTL loci (*qPH9-1*, *qLA5-1*, *qLA5-2*) identified in this study have not been reported in previous literatures. The QTL mapping results of this study have both similarities and differences with those of previous studies: the similarities confirm the reliability of the results of this study, and it is necessary to further perform fine mapping and even gene cloning for the loci overlapping with previous studies; the unreported new loci may be potential new QTLs related to plant architecture traits, which can provide a new direction for subsequent research in this field.The integrated BSA-F₂ strategy used in this study shows high efficiency and reliability in QTL mapping. BSA allows rapid preliminary mapping and quick identification of major QTL regions, while the F₂ population further validates the authenticity of these QTLs, thereby reducing false-positive results and ensuring accuracy for subsequent marker-assisted selection.

This strategy is more time-saving and convenient compared with traditional validation methods using multi-environment trials or secondary populations. In this study, using a strategy combining preliminary BSA mapping and F₂ segregating population validation, we identified five major plant architecture-related QTLs on chromosomes 5 and 9, showing a distribution feature of “QTL cluster on chromosome 5”—four QTLs *(qPH5-1*, *qTBN5-1*, *qLA5-1*, *qLA5-2*) were clustered on chromosome 5, while one PH QTL (*qPH9-1*) was located on chromosome 9. This distribution pattern suggests that chromosome 5 may harbor a gene cluster regulating plant architecture traits, providing a new perspective for dissecting the co-regulatory mechanisms of traits such as PH and LA. This finding provides valuable information and important implications for further genetic dissection and molecular improvement of maize plant architecture. The six candidate genes further screened are involved in key biological processes including cytoskeleton dynamics (ARP2/3 complex subunit ARPC3), meristematic stem cell homeostasis (protein phosphatase 2 C), transcriptional regulatory complex assembly (MED12 protein), cell cycle regulation (E2F transcription factor), and cell polarity establishment (SUN family protein containing Sad1/UNC domain), whose functions are closely related to the morphogenesis of plant architecture traits. For example, the ARP2/3 complex participates in cell elongation by regulating actin polymerization [45–51], and the E2F transcription factor affects the development of tassel branch primordia by regulating meristematic cell proliferation [53]. In plants, the actin cytoskeleton regulates cell elongation and affects organ growth [62]. *Zm00001eb238970* and *Zm00001eb238980* encode P21-Arc (ARPC3 subunit), and the ARP2/3 complex acts as a core regulator of the actin cytoskeleton [50]. Perturbation of actin polymerization or depolymerization significantly affects cell elongation and thus regulates plant height [63]. *Zm00001eb394120*, an ortholog of Arabidopsis *POL*, encodes protein phosphatase 2 C, which maintains the balance between stem cell proliferation and differentiation in meristems [64], and is essential for plant height development. *Zm00001eb243000* encodes an E2F transcription factor that regulates cell cycle and cell division [65]. Tassel branch number is determined by the activity and fate of inflorescence meristem and branch meristems [20]. Therefore, E2F may affect tassel branch development by regulating meristem cell proliferation. Asymmetric cell elongation in the leaf pulvinus leads to leaf bending and changes leaf angle [66]. SUN-domain proteins form the LINC complex with KASH proteins to connect the nucleoskeleton and cytoskeleton [67].

*Zm00001eb233650* encodes a SUN-domain protein, which may regulate leaf angle by modulating cytoskeleton organization, cell elongation and cell structure in the pulvinus. These results provide a solid foundation for further gene functional verification.

At the methodological level, the BSA technique rapidly narrows down candidate intervals through extreme phenotype bulks, significantly improving the efficiency of preliminary mapping [68].

However, its resolution is limited by the number of recombination events and is susceptible to phenotypic identification errors. In this study, we performed linkage analysis by constructing a 544-plant F₂ segregating population, which not only verified the reliability of the preliminary BSA mapping results but also refined major QTLs (e.g., *qPH5-1*) to a physical interval of 85.3–92.7 Mb, laying a foundation for subsequent map-based cloning. Although the F₂ population allowed the initial detection of QTLs for maize plant architecture traits, evaluation in only one environment represents a limitation. Single-environment trials cannot reveal genotype-by-environment interactions, which may affect QTL stability and lead to biased effect estimation. In addition, the F₂ population is a temporary segregating population that cannot be stably maintained for repeated tests. In future studies, we will validate these QTLs across multiple environments using advanced populations such as recombinant inbred lines (RILs) or near-isogenic lines (NILs).

High-density SNP markers will be used to saturate target genomic regions and achieve high-resolution fine mapping of major QTLs. Future research can focus on the following directions: (1) Develop high-density molecular markers in the intervals of major QTLs such as *qPH5-1* and *qLA5-2*, and achieve fine mapping and cloning of genes using recombinant inbred lines or near-isogenic lines; (2) In future research, functional verification of key candidate genes (e.g., *Zm00001eb238970*, *Zm00001eb233650*) will be conducted using CRISPR-Cas9 gene editing, EMS-induced mutants, or other transgenic approaches to clarify their biological functions and molecular mechanisms underlying maize plant architecture regulation; (3) Introduce major QTLs into elite maize inbred lines via molecular marker-assisted selection, and evaluate the effects of plant architecture improvement from aspects such as lodging resistance, photosynthetic efficiency, and pollination characteristics. In summary, the results of this study provide a theoretical basis for map-based cloning of major QTLs for maize plant architecture and their application in molecular breeding, and have important practical implications for promoting maize yield improvement.

## Conclusions

This study focused on the genetic regulatory mechanisms of maize plant architecture traits (PH, TBN, LA). Using BSA mapping combined with F₂ population validation, we identified five major QTLs on chromosomes 5 and 9, among which *qPH5-1* had the strongest effect on PH (explaining 13.75% of phenotypic variation, LOD = 19.87, additive effect=-15.31). Based on MaizeGDB annotations and functional predictions, six core candidate genes were identified in the QTL intervals. These findings provide a theoretical basis for map-based cloning of maize plant architecture QTLs and molecular marker-assisted breeding, and support maize high-yield architecture improvement. 

## Supplementary Information

Supplementary Material 1.

Supplementary Material 2.

## Data Availability

All data generated or analyzed during this study are included in this publishedarticle.

## Abbreviations

QTL: Quantitative Trait Locus
PH: Plant height
TBN: Tassel branch number
LA: Leaf angle
BSA: Bulked segregant analysis
LOD: Logarithm of odds
SSR: Simple sequence repeat
ICIM: Inclusive composite interval mapping
PLA: Penultimate leaf angle
AELA: Above-ear leaf angle
NCBI: National center for biotechnology information
SRA: Sequence read archive
